# Vector Competence of French Polynesian *Aedes aegypti* and *Aedes polynesiensis* for Zika Virus

**DOI:** 10.1371/journal.pntd.0005024

**Published:** 2016-09-21

**Authors:** Vaea Richard, Tuterarii Paoaafaite, Van-Mai Cao-Lormeau

**Affiliations:** Institut Louis Malardé, Papeete, Tahiti, French Polynesia; Fundaçao Oswaldo Cruz, BRAZIL

## Abstract

**Background:**

In 2013–2014, French Polynesia experienced for the first time a Zika outbreak. Two *Aedes* mosquitoes may have contributed to Zika virus (ZIKV) transmission in French Polynesia: the worldwide distributed *Ae*. *aegypti* and the Polynesian islands-endemic *Ae*. *polynesiensis* mosquito.

**Methodology/Principal Findings:**

To evaluate their vector competence for ZIKV, mosquitoes were infected *per os* at viral titers of 7 logs tissue culture infectious dose 50%. At several days post-infection (dpi), saliva was collected from each mosquito and inoculated onto C6/36 mosquito cells to check for the presence of ZIKV infectious particles. Legs and body of each mosquito were also collected and submitted separately to RNA extraction and ZIKV RT-PCR. In *Ae*. *aegypti* the infection rate was high as early as 6 dpi and the dissemination efficiency get substantial from 9 dpi while the both rates remained quite low in *Ae*. *polynesiensis*. The transmission efficiency was poor in *Ae*. *aegypti* until 14 dpi and no infectious saliva was found in *Ae*. *polynesiensis* at the time points studied.

**Conclusions/Significance:**

In our experimental conditions, the late ability of the French Polynesian *Ae*. *aegypti* to transmit ZIKV added by the poor competence of *Ae*. *polynesiensis* for this virus suggest the possible contribution of another vector for the propagation of ZIKV during the outbreak, in particular in remote islands where *Ae*. *polynesiensis* is predominating.

## Author Summary

From 2007, Zika virus has caused several outbreaks in the Pacific including French Polynesia. *Aedes aegypti* mosquito which is present in almost all Pacific Island Countries is reasonably expected to have been involved in the Zika outbreaks. In addition endemic *Aedes* mosquito species may have sustained Zika virus transmission in the less urbanized and most remote islands. In the present study we provide for the first time data about the vector competence of the endemic *Ae*. *polynesiensis* species for Zika virus. We found under experimental conditions a weak competence of *Ae*. *polynesiensis* for the virus. Furthermore we demonstrated a late ability of the French Polynesian population of *Ae*. *aegypti* to transmit Zika virus. These findings raise questions about the potential involvement of other vector(s) in Zika virus transmission in place or together with the *Aedes* mosquitoes. In a context where innovative vector control strategies are mostly focused on targeting the mosquito species considered as the main arbovirus vectors, the potential for others vector species to take the lead in transmitting such arboviruses should not be neglected.

## Introduction

Zika virus (ZIKV; *Flaviviridae*: *Flavivirus*) infection usually produces fever, skin rashes, conjunctivitis, muscle and joint pain, malaise and headache with potential neurological and auto-immune complications [1]. Isolated first in 1947 from a febrile monkey in the Zika forest in Uganda [2], only sporadic humans infections were reported in Africa and Asia since the first large outbreak appeared in 2007 in Yap Island, Federated States of Micronesia [3]. ZIKV is a single-stranded positive sense RNA virus. Three genetic lineages reflecting geographic origin have been described: the two original West and East African lineages; and the Asian lineage [4].

French Polynesia is a French overseas Territory of about 270 000 inhabitants located in the South Pacific Ocean. Until ZIKV emerged in October 2013, dengue virus (*Flaviviridae*: *Flavivirus*) used to be the only arbovirus recognized as circulating in French Polynesia [5]. ZIKV infections were reported in all five French Polynesian archipelagos (Society, Marquesas, Tuamotu, Gambier and Austral Islands) leading to the largest ZIKV outbreak ever reported at that date. Phylogenetic analysis determined that the virus belonged to the Asian lineage [5].

ZIKV has been described as being transmitted by peri-domestic *Aedes* mosquitoes, mostly *Ae*. *aegypti* but also *Ae*. *albopictus* that is able to survive at temperate climates [6–12]. Several other *Aedes* mosquito species have also been described as potential vectors for sylvatic transmission of ZIKV in Africa [8]. In the Pacific region, *Ae*. *aegypti* is present in almost all the region [13,14]. However in some islands where *Ae*. *aegypti* is absent or poorly present, endemic *Aedes* species like *Ae*. *hensilli* in Yap State predominate [15]. The potential role of *Ae*. *hensilli* in transmitting ZIKV during the outbreak in Yap island was suggested by its experimental ability to be infected and to disseminate the virus [15]. In French Polynesia the contribution of the endemic species *Ae*. *polynesiensis* in ZIKV spread was also suspected in addition of *Ae*. *aegypti*, supported by its ability to transmit arboviruses as DENV, chikungunya virus and Ross river virus [5,13,16,17].

In the present study, we assessed the vector competence of French Polynesian populations of *Ae*. *aegypti* and *Ae*. *polynesiensis* for ZIKV.

## Methods

### Virus

ZIKV strain PF13/251013-18 was isolated at Institut Louis Malardé from the serum of patient infected in October 2013 in the Marquesas Islands, French Polynesia. ZIKV was amplified on *Ae*. *albopictus* C6/36 cells [18] (ATCC CRL-1660, USA) as described in Richard et al. [19]. After three successive passages, the infected-cell supernatant was harvested and concentrated by using Centricon Plus-70 centrifugal filter devices (Millipore, Germany) [20]. ZIKV concentrate was supplemented with heat-inactivated foetal bovine serum (FBS, Life technologies, USA) at 1:5 and stored at -80°C.

For titrating the virus, C6/36 cells were inoculated with serial 10-fold dilutions of virus concentrate on a 96-wells plate. After six days, cells were fixed on the plate using 70% ice-cold acetone for 10 minutes. Cells were then incubated 45 minutes at 37°C with a specific hyperimmune mouse ascitic fluid provided by the Institut Pasteur of Dakar, Senegal, diluted 1:200 in PBS followed by 45 minutes of incubation at 37°C with fluorescein isothiocyanate-conjugated goat anti-mouse IgG (Bio-Rad Laboratories, France) diluted 1:100. Viral titers were evaluated with the method of Reed and Muench in 50% tissue culture infectious dose (TCID_50_/mL) [21].

### Mosquito Rearing

*Ae*. *aegypti* and *Ae*. *polynesiensis* colonies were established in 2014 from mosquito captured on Tahiti Island in Toahotu and Atimaono districts, respectively. F_16_ to F_18_ generation eggs of each mosquito colony were hatched. Larvae, pupae and adult mosquitoes were reared at 27°C, 80% relative humidity and 12:12h light-dark cycle inside a climate chamber (Sanyo MLR-351H, Japan) as previously described [19].

### Mosquito Infection

Five-days-old mosquitoes were starved for 24 hours and transferred into containers of about 60 mosquitoes each. The infectious meal was prepared using fresh washed bovine red cells (SAEM Abattage de Tahiti, French Polynesia), viral concentrate (1:29) and adenosine triphosphate (A6419, Sigma-Aldrich, USA) at 5 mM as phagostimulant. The titer of ZIKV in the blood meal was 7 log_10_ TCID_50_/mL. The blood meal was maintained at 37°C and presented during one hour through a Parafilm-M membrane to *Ae*. *aegypti* and through a porcine membrane to *Ae*. *polynesiensis*. To avoid horizontal transmission during sugar-feeding, fully-engorged females were transferred into individual plastic containers [22,23]. Mosquitoes were given access to 10% sucrose solution and maintained at 27°C, 80% relative humidity and 12:12h light-dark cycle for up to 21 days.

### Saliva Collection

Subsets of 18 hours starved mosquitoes were cold-anesthetized at 2, 6, 9, 14 and 21 days post-infection (dpi). To collect the saliva from each mosquito, legs and wings were removed and the proboscis was inserted into a filter tips ART (Molecular BioProducts, USA) containing 20 μL of FBS. Mosquitoes were invited to expectorate saliva during 30 min. The collected saliva was then expelled into a microtube containing 80 μL of 1% FBS cell-culture medium and preserved at -80°C. Each saliva sample was inoculated to C6/36 cells on a 96-well plate. Six days later, infectious cells were identified by indirect immunofluorescent assay as described above.

Body (thorax and abdomen) and legs from each mosquito were also preserved in separate microtubes at -80°C.

### RNA Extraction and Reverse Transcription Polymerase Chain Reaction (RT-PCR)

Individual mosquito bodies and legs were homogenized with metal beads for 4 min at 20 Hz (Mixer Mill Retsch MM301, Germany) using cell-culture medium supplemented at 20% FBS for bodies and NucliSENS lysis buffer (bioMérieux, France) for legs [19]. Homogenate supernatants were recovered after centrifugation 5 minutes at 20,000 x g. Nucleic acids were extracted with the NucliSENS miniMAG system (bioMérieux, France) in accordance with manufacturer’s instructions. Real time RT-PCR were performed on a CFX96 Touch Real-Time PCR Detection System instrument using iTaq Universal Probes One-Step Kit (Bio-Rad Laboratories, France) and the primers and probes previously described [4].

### Data and Statistical Analysis

Vector competence is defined as the ability of a given mosquito to allow the virus replicating, disseminating and finally being transmitted to a new susceptible host [24]. The ability of a mosquito species to become infected by the virus was given by the mosquito infection rate that corresponds to the proportion of females for which ZIKV was detected by real time RT-PCR in the body. The ability of the mosquito species to allow the virus spread outside the midgut was based on the detection of ZIKV in legs by real time RT-PCR. The evidence for the mosquito species to be able to transmit infectious virus was provided by the detection of replicative ZIKV particles from mosquito saliva inoculated on C6/36 cells. Viral dissemination and transmission efficiencies were defined as the number of mosquitoes with positive legs or infectious saliva divided by the number of mosquitoes tested.

As the mortality rate of *Ae*. *polynesiensis* in laboratory conditions was high (S1 Table), several trials were performed to obtain a sufficient number of mosquitoes for the different collecting days and especially for the late time point 14 dpi. The results obtained for each trial are detailed in S2 Table. Data of the trials performed with *Ae*. *polynesiensis* were pooled together before being analyzed.

Chi-square test with or without Yates’ correction or Fisher’s exact test were performed to evaluate the differences between the two *Aedes* species at each time point and between two successive time points for each species (Graph Pad Prism software, USA).

## Results

The mosquito infection rate was assessed at 6, 9, 14 and 21 dpi. To prevent any false positives due to remaining infectious blood meal in the midgut, infection rate was not evaluated at 2 dpi. The infection rate was 90% as early as 6 dpi for *Ae*. *aegypti* and increased slowly for *Ae*. *polynesiensis* from 11% at 6 dpi to 36% at 14 dpi (Table 1). The infection rate was significantly higher for *Ae*. *aegypti* compared to *Ae*. *polynesiensis* from 6 dpi to 14 dpi (p<0.0001).

**Table 1:** ZIKV infection rate, dissemination and transmission efficiencies in *Ae*. *aegypti* and *Ae*. *polynesiensis* from French Polynesia.

		2 dpi	6 dpi	9 dpi	14 dpi	21 dpi
**% of infection** (Number of infected bodies / number of mosquitoes tested)	***Ae*. *aegypti***	nd	90% (35/39)	93% (37/40)	85% (33/39)	93% (37/40)
	***Ae*. *polynesiensis***	nd	11% (10/95) ****	20% (18/89) ****	36% (24/66) ****	-
**% of dissemination** (Number of infected legs / number of mosquitoes tested)	***Ae*. *aegypti***	0% (0/39)	18% (7/39)	75% (30/40)	85% (33/39)	93% (37/40)
	***Ae*. *polynesiensis***	0% (0/77)	0% (0/95) ***	3% (3/89) ****	18% (12/66) ****	-
**% of transmission** (Number of infectious saliva / number of mosquitoes tested)	***Ae*. *aegypti***	0% (0/39)	3% (1/39)	8% (3/40)	36% (14/39)	73% (29/40)
	***Ae*. *polynesiensis***	0% (0/77)	0% (0/95)	0% (0/89) *	0% (0/66) ****	-

Infection and dissemination were determined by real-time RT-PCR. Transmission was evaluated by inoculation of saliva on C6/36 cells to detect infectious particles of ZIKV. At 2 days post-infection, the number of infected bodies was not determined (nd) due to remaining blood-meal in midgut. Statistically significant differences between the two species at each time point are shown by asterisks (* = p<0.05; *** = p<0.001; **** = p<0.0001). A dash (-) indicates that mosquitoes were not obtained for this collecting day post-infection (dpi).

**Fig 1:**
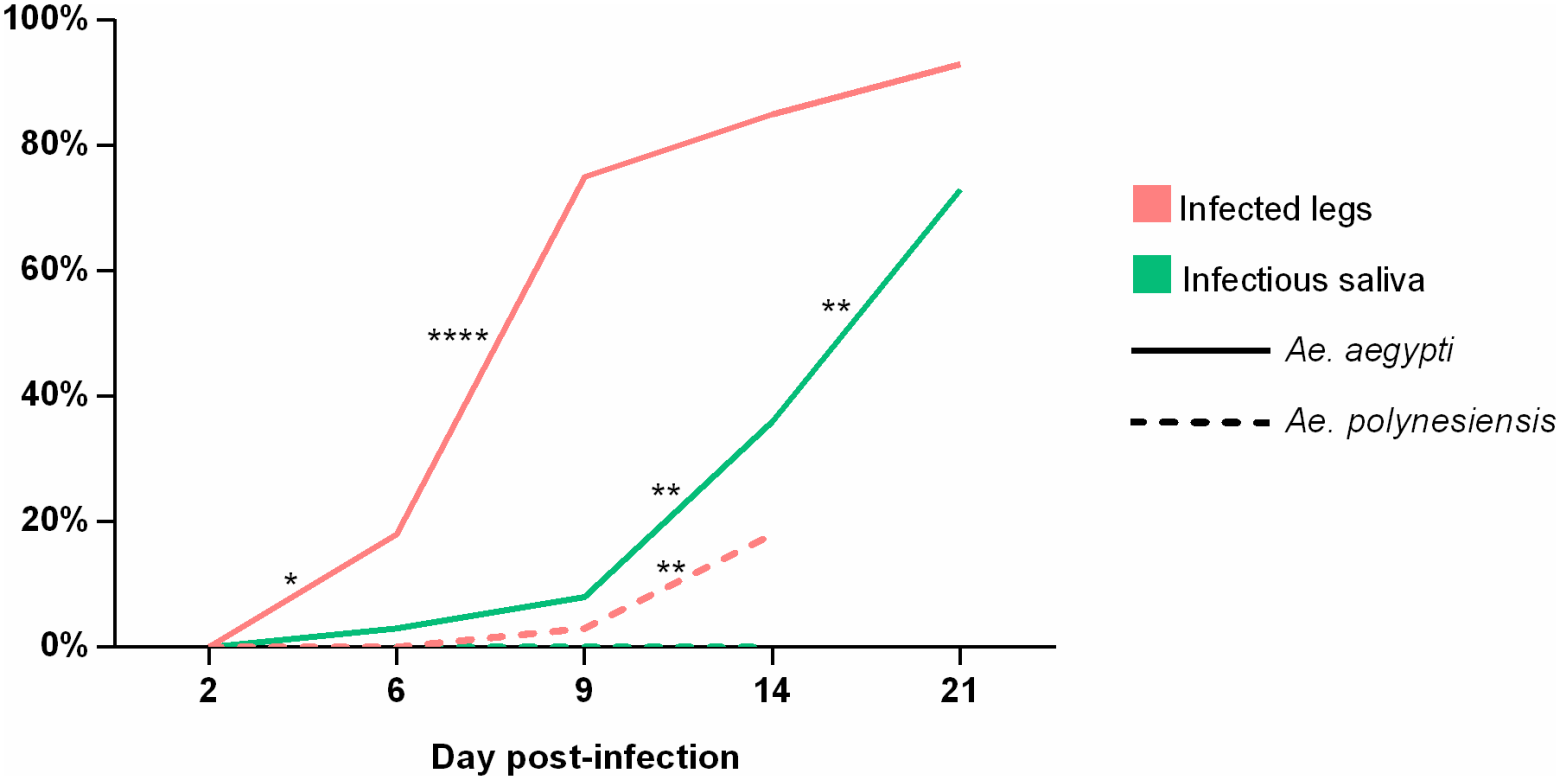
Progression trends of ZIKV dissemination and transmission efficiencies in *Ae*. *aegypti* and *Ae*. *polynesiensis*. Statistically significant differences between two successive days post-infection are indicated by asterisks (* = p<0.05; ** = p<0.01; **** = p<0.0001).

At 2 dpi, no ZIKV was detected in legs from *Ae*. *aegypti* and *Ae*. *polynesiensis* mosquitoes (Table 1). In *Ae*. *aegypti*, the dissemination efficiency increased over days, especially between 6 and 9 dpi (p<0.0001; Fig 1) getting 75% at 9 dpi. We noticed that at 14 and 21 dpi, the dissemination efficiency was equal to the infection rate indicating that all infected mosquitoes had disseminated the virus (Table 1). In *Ae*. *polynesiensis* mosquitoes, ZIKV appeared in legs at only 9 dpi with 3% of dissemination efficiency (Table 1). Despite a significant increase (p<0.01; Fig 1) the dissemination efficiency did not exceed 18% at 14 dpi. The dissemination rate in legs was significantly higher for *Ae*. *aegypti* compared to *Ae*. *polynesiensis* (p<0.001 at 6 dpi, p<0.0001 at 9 and 14 dpi; Table 1).

Infectious saliva was detected from 6 dpi in *Ae*. *aegypti* females with 3% of transmission efficiency (Table 1). The transmission efficiency remained low at 9 dpi (8%) then significantly increased to reach 36% at 14 dpi and 73% at 21 dpi (p<0.01; Fig 1). No ZIKV particle was found in the saliva from *Ae*. *polynesiensis* at any collecting days (Table 1).

## Discussion

In the present study we observed that *Ae*. *aegypti* early displayed high ZIKV infection rate but late ability to transmit the virus in our experimental conditions. Our results contrast with those previously reported for *Ae*. *aegypti* from Singapore in which as early as 6 dpi 100% of mosquitoes were potentially infectious with ZIKV detected in the salivary glands [10]. Contrariwise our results are in accordance with the recent studies carried in Senegalese and American populations of *Aedes* species [8,12]. Such a long extrinsic incubation period may limit the time window for an infectious vector to transmit ZIKV to susceptible people. Our laboratory results support that *Ae*. *aegypti* may have been a vector of ZIKV during the outbreak in French Polynesia, but maybe not the only one. In the endemic species *Ae*. *polynesiensis*, we found a moderate infection rate and the dissemination efficiency was low. No ZIKV particle was found in *Ae*. *polynesiensis* mosquito saliva even at the latest time point available for this species, i.e. 14 dpi. Due to the slower progression of ZIKV in this endemic species, the viral amount was maybe too low to allow detection at this time point. It is likely that ZIKV particles would have been detected in *Ae*. *polynesiensis* saliva at later time points. In a previous study, we had also observed that the dissemination and transmission of chikungunya virus in *Ae*. *polynesiensis* progressed slower than in *Ae*. *aegypti* [19].

The late ability of the two mainly distributed *Aedes* mosquito species in French Polynesia to transmit ZIKV raises questionings on the sole involvement of these vectors for having sustained the Zika outbreak, in particular in remote areas where *Ae*. *aegypti* is not or poorly present. In their study, Diagne et al. highlighted that the low transmission rates found in several African *Aedes* species are difficult to reconcile with continuous ZIKV transmission observed in Africa and also suggested the involvement of others vectors [8]. In Yap island, the predominant species *Ae*. *hensilli* was shown as a potential vector of ZIKV [15]. Nevertheless, despite a high level of infection (90%) at 8 dpi, only 20% of infected mosquitoes disseminated the virus, while for chikungunya virus the dissemination rate reached 80% for an infection rate of 60% [15]. Interestingly, in the same study *Ae*. *aegypti* was only found at 0,1% on the island while *Culex quinquefasciatus* was found at ~30% making it the second main species on the island. *Culex* species and notably *Culex quinquefasciatus* are also present in all five archipelagos of French Polynesia [25]. The potential role of *Culex* species in ZIKV spreading was recently suggested in a few communications [12,26,27].

On 1^st^ February 2015, subsequent to the report of an increase of microcephaly cases and neurological complications, including Guillain-Barré Syndrome, in ZIKV affected countries, the World Health Organization declared ZIKV a Public Health Emergency of International Concern. Zika is asymptomatic in most cases and no vaccine is already available, thus vector control remains essential to limit outbreaks and consequently to limit the occurrence of clinical cases with pathologic complications. Identifying the main vector(s) for the current pandemic strain of ZIKV is essential to properly adapt vector control strategies.

## Supporting Information

S1 Table Mortality rate from the day of infection to 21 dpi. The mortality no exceeded 2% from 2 to 21 dpi for *Ae*. *aegypti* while for *Ae*. *polynesiensis* it was ~30% on the 2–9 dpi period and reached more than 70% during the 9–14 dpi period. N, number of females allowed feeding on ZIKV infectious blood-meal; n, number of females remaining from the previous period minus the number of females sacrificed for testing on the previous sampling day; dpi, days post-infection. A dash (-) indicates there was no more female at these collecting days.
(DOCX)

S2 Table Number of infected bodies, infected legs and infectious saliva obtained in each experimental trial performed with *Ae*. *polynesiensis* mosquitoes. At 2 days post-infection, the number of ZIKV infected bodies was not determined (nd) due to remaining blood-meal in midgut. A dash (-) indicates that mosquitoes were not collected at these days post-infection. n, number of mosquitoes collected; dpi, days post-infection. Infection rates and dissemination efficiencies obtained in each trial are indicated in brackets. For each time point, the rates were not significantly different between trials (Chi-square test with or without Yates’ correction or Fisher’s exact test; Graph Pad Prism software, USA).
(DOCX)
