# Supplementary material for: Vector Competence of French Polynesian Aedes aegypti and Aedes polynesiensis for Zika Virus
Source: PLoS Negl Trop Dis. 2016 Sep 21;10(9):e0005024. doi: 10.1371/journal.pntd.0005024 (PMC5031459; doi:10.1371/journal.pntd.0005024)
Supplement: S1 Table — The mortality no exceeded 2% from 2 to 21 dpi for Ae. aegypti while for Ae. polynesiensis it was ~30% on the 2–9 dpi period and reached more than 70% during the 9–14 dpi period. N, number of females allowed feeding on ZIKV infectious blood-meal; n, number of females remaining from the previous period minus the number of females sacrificed for testing on the previous sampling day; dpi, days post-infection. A dash (-) indicates there was no more female at these collecting days. (DOCX) [file pntd.0005024.s001.docx]

**S1 Table. Mortality rate from the day of infection to 21 dpi.** The mortality no exceeded 2% from 2 to 21 dpi for *Ae. aegypti* while for *Ae. polynesiensis* it was ~30% on the 2-9 dpi period and reached more than 70% during the 9-14 dpi period. N, number of females allowed feeding on ZIKV infectious blood-meal; n, number of females remaining from the previous period minus the number of females sacrificed for testing on the previous sampling day; dpi, days post-infection. A dash (-) indicates there was no more female at these collecting days.

|  | | **Number of engorged females / N (% of engorged females)** | **Number of dead females / n (% of mortality)** | | | | |
| --- | --- | --- | --- | --- | --- | --- | --- |
|  |  |  | **0-2 dpi** | **2-6 dpi** | **6-9 dpi** | **9-14 dpi** | **14-21 dpi** |
| ***Ae. polynesiensis*** | **Trial 1** | 163/507  (32%) | 20/163  (12%) | 83/106  (78%) | - | - | - |
|  | **Trial 2** | 153/468  (33%) | 9/153  (6%) | 32/144  (22%) | 16/75  (21%) | - | - |
|  | **Trial 3** | 244/730  (33%) | 22/244  (9%) | 60/182  (33%) | 35/87  (40%) | - | - |
|  | **Trial 4** | 167/484  (34%) | 37/167  (22%) | 43/130  (33%) | 29/87  (33%) | 44/58  (76%) | - |
|  | **Trial 5** | 180/498  (36%) | 6/180  (3%) | 50/174  (29%) | 51/124  (41%) | 51/73  (70%) | - |
|  | **Trial 6** | 229/709  (32%) | 11/229  (5%) | 63/218  (29%) | 34/155  (22%) | 91/121  (75%) | - |
|  | **Total** | 1136/3396  (33%) | 105/1136  (9%) | 331/954  (35%) | 165/528  (31%) | 186/252  (74%) | - |
| ***Ae. aegypti*** | | 219/282  (78%) | 1/219  (0.5%) | 3/179  (1.7%) | 0/137  (0%) | 1/97  (1%) | 1/57  (1.7%) |
