# Supplementary material for: Vector Competence of French Polynesian Aedes aegypti and Aedes polynesiensis for Zika Virus
Source: PLoS Negl Trop Dis. 2016 Sep 21;10(9):e0005024. doi: 10.1371/journal.pntd.0005024 (PMC5031459; doi:10.1371/journal.pntd.0005024)
Supplement: S2 Table — At 2 days post-infection, the number of ZIKV infected bodies was not determined (nd) due to remaining blood-meal in midgut. A dash (-) indicates that mosquitoes were not collected at these days post-infection. n, number of mosquitoes collected; dpi, days post-infection. Infection rates and dissemination efficiencies obtained in each trial are indicated in brackets. For each time point, the rates were not significantly different between trials (Chi-square test with or without Yates’ correction or Fisher’s exact test; Graph Pad Prism software, USA). (DOCX) [file pntd.0005024.s002.docx]

**S2 Table. Number of infected bodies, infected legs and infectious saliva obtained in each experimental trial performed with *Ae. polynesiensis* mosquitoes.** At 2 days post-infection, the number of ZIKV infected bodies was not determined (nd) due to remaining blood-meal in midgut. A dash (-) indicates that mosquitoes were not collected at these days post-infection. n, number of mosquitoes collected; dpi, days post-infection. Infection rates and dissemination efficiencies obtained in each trial are indicated in brackets. For each time point, the rates were not significantly different between trials (Chi-square test with or without Yates’ correction or Fisher’s exact test; Graph Pad Prism software, USA).

|  | | **Trial 1** | **Trial 2** | **Trial 3** | **Trial 4** | **Trial 5** | **Trial 6** | **Total** |
| --- | --- | --- | --- | --- | --- | --- | --- | --- |
| **2 dpi** | **n** | 37 | - | 40 | - | - | - | **77** |
|  | **Infected bodies** | nd | - | nd | - | - | - | **nd** |
|  | **Infected legs** | 0 | - | 0 | - | - | - | **0** |
|  | **Infectious saliva** | 0 | - | 0 | - | - | - | **0** |
| **6 dpi** | **n** | 23 | 37 | 35 | - | - | - | **95** |
|  | **Infected bodies** | 2 (9%) | 4 (11%) | 4 (11%) | - | - | - | **10** |
|  | **Infected legs** | 0 | 0 | 0 | - | - | - | **0** |
|  | **Infectious saliva** | 0 | 0 | 0 | - | - | - | **0** |
| **9 dpi** | **n** | - | 37 | 52 | - | - | - | **89** |
|  | **Infected bodies** | - | 6 (16%) | 12 (23%) | - | - | - | **18** |
|  | **Infected legs** | - | 1 (3%) | 2 (4%) | - | - | - | **3** |
|  | **Infectious saliva** | - | 0 | 0 | - | - | - | **0** |
| **14 dpi** | **n** | - | - | - | 14 | 22 | 30 | **66** |
|  | **Infected bodies** | - | - | - | 5 (36%) | 8 (36%) | 11 (37%) | **24** |
|  | **Infected legs** | - | - | - | 2 (14%) | 5 (23%) | 5 (17%) | **12** |
|  | **Infectious saliva** | - | - | - | 0 | 0 | 0 | **0** |
